# The increasing prevalence of CPV-2c in domestic dogs in China

**DOI:** 10.7717/peerj.9869

**Published:** 2020-09-29

**Authors:** Xiangqi Hao, Yuwei He, Chuhan Wang, Weiqi Xiao, Ruohan Liu, Xiangyu Xiao, Pei Zhou, Shoujun Li

**Affiliations:** 1 Key Laboratory of Comprehensive Prevention and Control for Severe Clinical Animal Diseases of Guangdong Province, College of Veterinary Medicine, South China Agricultural University, Guangzhou, Guangdong Province, China; 2 State Key Laboratory of Veterinary Biotechnology, Harbin Veterinary Research Institute, Chinese Academy of Agricultural Sciences, Harbin, China; 3 Guangdong Laboratory for Lingnan Modern Agriculture, Guangzhou, China; 4 Guangdong Engineering and Technological Research Center for Pets, College of Veterinary Medicine, South China Agricultural University, Guangzhou, Guangdong Province, China

**Keywords:** CPV-2c, VP2, Aetiological survey, Prevalence

## Abstract

**Background:**

Canine parvovirus type 2 (CPV-2), a serious pathogen, leads to high morbidity and mortality in dogs and several wild carnivore species. Although it is a DNA virus, it evolves particularly rapidly, with a genomic substitution rate of approximately 10^−4^ substitutions/site/year, close to that of some RNA viruses. Tracing the prevalence of CPV-2 in dogs is significant.

**Methods:**

In this study, an aetiological survey was carried out from 2016 to 2019 in Guangdong Province, China, involving Guangzhou, Shenzhen and Dongguan. Furthermore, to systematically analyse the prevalence of CPV-2 in China, the VP2 gene sequences of all Chinese isolates were downloaded from the NCBI nucleotide database in December 2019, and changes in CPV-2 variants were examined.

**Results:**

A total of 55.7% (34/61) of samples were CPV-2 positive by PCR detection and virus isolation. In addition to different variants circulating in dogs, coinfection with multiple variants was identified, as was coinfection with other canine enteric pathogens in some cases. Two previously reported amino acid sites, A5G and Q370R of CPV-2c mutants, reported in variants in China were assessed, and several CPV-2 isolates with P13S and K582N mutations were detected in this study. Finally, we speculate on the prevalence of different CPV-2 variants in China. According to the VP2 gene sequence obtained from the NCBI nucleotide database, the proportion of different variants in China has changed, and CPV-2c appears to be growing rapidly. In conclusion, this aetiology survey suggests that CPV-2 continues to be common in China and that the prevalence of CPV-2c is increasing.

## Introduction

Canine parvovirus type 2 (CPV-2), which belongs to the genus *Protoparvovirus*, family *Parvoviridae*, is an important pathogen that leads to high morbidity and mortality in several wild carnivore species and even non-carnivores (Appel, Scott & Carmichael, 1979; Burtonboy et al., 1979; Nelson et al., 1979; Pletcher et al., 1979; Wang et al., 2020). CPV-2 is a non-enveloped virus, with a single-stranded DNA genome encoding 2 large open reading frames (ORFs). VP2, the main capsid protein of the virus, is involved in determining viral tissue orientation and host range (Hueffer et al., 2003; Nelson et al., 2007). NS1, a versatile phosphorylated protein, facilitates DNA packaging and viral replication (Gupta et al., 2016; Saxena et al., 2013).

CPV-2 was considered to be a change in host range due to mutations in the feline panleukopenia virus (FPV) (Appel, Scott & Carmichael, 1979; Black et al., 1979; Johnson & Spradbrow, 1979; Osterhaus, Steenis & Kreek, 1980; Parrish, 1990), and it has become global since its discovery in the 1970s (Appel et al., 1979; Appel & Carmichael, 1980; Carmichael & Binn, 1981). Subsequently, CPV-2a and CPV-2b, two new variants, have emerged and replaced the initial CPV-2 (Parrish et al., 1991; Perez et al., 2012). CPV-2c, a newer variant, was later found in Italy (Buonavoglia et al., 2001) and spread rapidly worldwide (Decaro & Buonavoglia, 2012; Decaro et al., 2005; Decaro et al., 2006; Joao Vieira et al., 2008; Kapil et al., 2007; Nakamura et al., 2004; Nandi et al., 2010; Perez et al., 2007; Puentes et al., 2012; Streck et al., 2009; Sutton et al., 2013; Touihri et al., 2009). Notably, the original CPV-2 cannot infect cats (Truyen et al., 1996); however, the strains, CPV-2a, CPV-2b and CPV-2c, not only susceptible to dogs but also circulate in cats. Consequently, these variants have become an essential pathogen for feline panleukopenia disease (Ikeda et al., 2000; Truyen et al., 1996). Since the emergence of the different strains, research on their associated detailed amino acid mutations has been inconsistent. According to the latest report on the VP2 gene (Zhou et al., 2017), six aa mutations (K80R, K93N, V103A, D323N, N564S, A568G) are present in CPV compared with FPV, with three or four aa mutations (M87 L, A300G/D, D305Y and I101T not sure) in CPV-2a compared with CPV-2. However, compared with CPV-2a, only one mutation residue (N426D) was observed in CPV-2b, and D426E was found in CPV-2c compared with CPV-2b (Miranda & Thompson, 2016). To trace the prevalence of CPV-2 in domestic dogs in Guangdong Province, China, an aetiological survey was carried out from 2016 to 2019.

CPV-2a, CPV-2b and CPV-2c co-circulate in most regions on a global scale. As previously noted, CPV-2a exists in most areas in Asia (Zhou et al., 2017); it is worth noting that CPV-2c is also present in some Asian regions (Charoenkul et al., 2019; Vannamahaxay et al., 2017). Although CPV-2c is identified as the dominant strain in Europe and Latin America (Hong et al., 2007; Tucciarone et al., 2018; Zhou et al., 2017), it has gradually become the dominant variant in Laos, Vietnam, Thailand and other regions (Charoenkul et al., 2019; Lin et al., 2017; Vannamahaxay et al., 2017). CPV-2c was reported in China in 2010 (Zhang et al., 2010a; Zhang et al., 2010b), but two recent studies reported CPV-2a as the major strain in dogs in China (Zhao et al., 2017; Zhuang et al., 2019). Regardless, based on the evolution of parvovirus in other Asian regions, it is clear that this is only a temporary phenomenon and that CPV-2c will be isolated in an increasing number of locations (Li et al., 2019; Lin et al., 2017). Therefore, the prevalence of CPV-2 in China needs to be re-studied.

Since CPV-2 was reported, it has been circulating in the dog population, despite the development of related vaccines and the certain efficacy of the vaccine in preventing CPV disease. Indeed, CPV-2 mutates constantly, and new variants are continuously produced. Moreover, immune failure occurs on occasion, and the prevention and treatment of parvovirus disease is very serious (De et al., 2018; Wu et al., 2015). In particular, the evolution of VP2 deserves attention, as genetic surveillance of circulating viruses is essential for the prevention and control of CPV infection and the development of more effective vaccines.

## Materials & Methods

### Sample collection

Between 2016 and 2019, a total of 61 stool samples were collected for testing, as obtained from 61 domestic dogs with parvovirus infection-like clinical signs. The dogs were taken to the hospital by their respective owners at different times, and each stool sample was collected with their owner’s verbal permission. No animal experiments were conducted in this study, and only stool samples were collected. The veterinary clinics that provided samples of sick dogs were located in Guangzhou, Shenzhen and Dongguan. After the samples were collected, they were placed at −80 °C.

### PCR detection for CPV-2 and other canine enteric pathogens

Genomic DNA was extracted from samples using a commercial RaPure Viral RNA/DNA Kit (R4410-02, Magen, Guangzhou). The PCR primers and procedures used for detection were referenced from previous studies (Mittal et al., 2014). PCR products of approximately 567 bp were considered positive.

CPV-2-positive faecal samples were also detected for canine norovirus (CNoV), canine astrovirus (CaAstV), canine kobuvirus (CaKV), canine bocavirus (CBoV), canine coronavirus (CCoV), and group A-rotavirus (RV-A) by either PCR or reverse transcription-PCR (RT-PCR). The RNA extracted from CPV-2-positive samples was reverse transcribed to cDNA using random primers (TaKaRa, Japan) and Moloney murine leukaemia virus (M-MLV) reverse transcriptase (TaKaRa, Japan) according to the manufacturer’s instructions. The amplification procedure was described in previous studies (Costa et al., 2014; Di Martino et al., 2013; Geng et al., 2015; Gomez et al., 2011; Grellet et al., 2012; Lau et al., 2012; Mesquita & Nascimento, 2012).

### Virus isolation

Each PCR-positive sample of CPV-2 was inoculated onto a monolayer of feline kidney (F81) cells (Provided by the Chinese Academy of Sciences Cell Bank) for 5 days. Then, each culture medium was centrifuged at 12,000 rpm for 10 min at 4 °C, and each supernatant was harvested and stored at −80 °C.

### VP2 sequencing and analysis

The primers used to amplify VP2 genes were CPV-VP2-F (5′- ATGAGTGATGGAGC AGTTCAACCA-3′) and CPV-VP2-R (5′-TTAGTATAATTTTCTAGGTGCTA G-3′). The amplification procedure was as follows: 35 cycles of 95 °C for 30 s, 53 °C for 30 s, and 72 °C for 2 min. The PCR products were purified using an Agarose Gel DNA purification kit (Magen, Guangzhou) and sequenced using synthetic oligo nucleotides from Invitrogen. If there were double peaks in the sequences, the PCR products were ligated to the pMD18-T simple vector, and several clones were sequenced to identify each genome. To construct a phylogenetic tree, other sequences, such as FPV, mink enteritis virus (MEV), raccoon parvovirus (RPV), and blue fox parvovirus (BFPV) were also downloaded from the NCBI website, and they were selected as outgroups to determine the tree roots. Gene sequences of VP2 were aligned with BioEdit (Version 7.0.9.0) (Hall, 2011). All phylogenetic trees were constructed with MEGA software (Version 7.0.26) (Tamura et al., 2011). First, the tree was constructed with the neighbour-joining method, with 1,000 bootstraps. Then, the virus sequences from the neighbour-joining trees were selected, and the maximum likelihood method (Kimura 2-parameter model) was employed to reconstruct the trees (bootstrap replicates = 1,000).

### The latest prevalence of the CPV-2 variant in China

To systematically analyse the prevalence of CPV-2, the VP2 gene sequences of all Chinese isolates were downloaded from the NCBI nucleotide database in December 2019. On the basis of annotating specific collection dates, complete sequences, partial coding region sequences with descriptions of variant identification, and 38 sequences isolated in this study were also selected. Ultimately, all 1076 Chinese CPV-2 isolates were classified and analysed according to collection date and variant type. Graphs were created using Prism 6.01 software (GraphPad Software).

## Results

### Investigation of CPV-2 and other enteric pathogens

Sixty-one domestic dogs were tested. Based on PCR results, 55.7% (34/61) of the samples contained CPV-2, and isolates were obtained by inoculation into F81 cells. CPV-2 coinfection with other pathogens was found: two samples (samples 3 and 5) presented coinfection with CaAstV; one sample (sample 6) coinfection with CCoV; and four samples (samples 8, 16, 22 and 23) coinfection with CaKV (Table 1).

**Table 1:** Virus infection characteristics of the CPV-2-positive dogs.

Sample	Viruses No.	GenBank accession No.	Strain name	Variants	Coinfection with variants	Coinfection with other viruses
1	1	KY937668	canine/Guangzhou/P1-1/2016	CPV-2b	No	/
	2	KY937669	canine/Guangzhou/P1-2/2016	CPV-2a	No	/
2	3	KY937670	canine/Guangzhou/P2/2016	CPV-2b	No	/
3	4	KY937671	canine/Guangzhou/P3/2016	CPV-2a	Yes	CaAstV
4	5	KY937672	canine/Guangzhou/P5/2016	CPV-2a	No	/
5	6	KY937674	canine/Guangzhou/T/2016	CPV-2a	Yes	CaAstV
6	7	KY968642	canine/Guangzhou/Z1/2016	CPV-2b	Yes	CCoV
7	8	KY968643	canine/Guangzhou/Z2/2016	CPV-2b	No	/
8	9	KY937656	canine/Guangzhou/F2/2016	CPV-2b	Yes	CaKV
9	10	KY937657	canine/Guangzhou/F3-1/2016	CPV-2b	No	/
	11	KY937658	canine/Guangzhou/F3-2/2016	CPV-2c	No	/
10	12	KY937650	canine/Guangzhou/B/2016	CPV-2c	No	/
11	13	KY937647	canine/Shenzhen/11-1/2016	CPV-2c	No	/
	14	KY937648	canine/Shenzhen/11-2/2016	CPV-2c	No	/
12	15	KY937662	canine/Shenzhen/H1/2016	CPV-2c	No	/
13	16	KY937663	canine/Shenzhen/H4/2016	CPV-2b	No	/
14	17	KY937675	canine/Shenzhen/Y3/2016	CPV-2a	No	/
15	18	KY937673	canine/Shenzhen/S1/2016	CPV-2a	No	/
16	19	KY937660	canine/Guangzhou/GZ-4/2017	CPV-2a	Yes	CaKV
17	20	KY937659	canine/Guangzhou/GZ-1/2017	CPV-2b	No	/
18	21	KY937661	canine/Guangzhou/GZ-5/2017	CPV-2a	No	/
19	22	MT488467	canine/Guangzhou/DH1130-1/2017	CPV-2c	No	/
	23	MT488468	canine/Guangzhou/DH1130-2/2017	CPV-2c	No	/
20	24	MT488454	canine/Guangzhou/CWC/2018	CPV-2c	No	/
21	25	MT488456	canine/Guangzhou/QH1/2018	CPV-2a	No	/
22	26	MT488457	canine/Guangzhou/QH2/2018	CPV-2a	Yes	CaKV
23	27	MT488458	canine/Guangzhou/QH3/2018	CPV-2c	Yes	CaKV
24	28	MT488459	canine/Guangzhou/QH5/2018	CPV-2c	No	/
25	29	MT488460	canine/Dongguan/XH1/2018	CPV-2c	No	/
26	30	MT488461	canine/Dongguan/XH2/2018	CPV-2a	No	/
27	31	MT488462	canine/Dongguan/XH3/2018	CPV-2a	No	/
28	32	MT488463	canine/Shenzhen/XH4/2018	CPV-2a	No	/
29	33	MT488464	canine/Shenzhen/XH5/2018	CPV-2a	No	/
30	34	MT488465	canine/Guangzhou/DH6/2018	CPV-2c	No	/
31	35	MT488466	canine/Guangzhou/DH7/2018	CPV-2b	No	/
32	36	MT488455	canine/Guangzhou/QG3/2019	CPV-2c	No	/
33	37	MT488453	canine/Guangzhou/QG18/2019	CPV-2c	No	/
34	38	MT488452	canine/Guangzhou/QG21/2019	CPV-2c	No	/

**Notes.**

/ No viruses

The VP2 gene was amplified among the 34 isolates, and several double peaks in the sequences of four samples were observed. Therefore, the PCR products of these four samples were ligated to the pMD18-T simple vector, and several clones were selected and sequenced for each genome. Among them, two different variants (CPV-2a and CPV-2b as well as CPV-2b and CPV-2c) of two viral genomes were identified in two samples; additionally, the same variant (CPV-2c) of two viral genomes was identified in two samples (Table 1). Finally, 38 viral VP2 sequences were amplified from 34 isolates. In our research, 36.84% (14/38) of the variants were CPV-2a, 23.68% (9/38) were CPV-2b, and 39.47% (15/38) were CPV-2c. In our previous study, CPV-2a was found to be the main variant in Asia (Zhou et al., 2017), but the proportions of CPV-2c exceed those of CPV-2a in recent aetiological investigations in Guangdong.

### Amino acid analysis

Mutation of the VP2 is a crucial aspect of virus evolution and is significant in distinguishing different variants. The key aa sites for virus evolution are summarized in Table 2. The substitution tendency of F267Y, Y324I, and T440A has been emphasized in previous studies (Chinchkar et al., 2006; Decaro & Buonavoglia, 2012; Zhou et al., 2017). A5G and Q370R, which were recently reported in China, are thought to be unique CPV-2c mutation sites related to infection and pathogenicity (Geng et al., 2015; Wang et al., 2016b). These variants were detected in only a portion of the CPV-2c strains. In addition to these two sites, a novel mutation in VP2 of CPV-2c was found at residue 13, P13S, which appeared in multiple strains. Mutations in K582N were also present in three strains. In this study, 5.26% (2/38) of isolates presented 5G, 15.79% (6/38) presented 13S, 100% (38/38) presented 267Y, 100% (38/38) presented 324I, 28.95% (11/38) presented 370R and 57.89% (22/38) presented 440A.

**Table 2:** The amino acid characteristic of VP2.

**Viruses No.**	**GenBank accession No.**	**Strain name**	**Variants**	**5**	**13**	**267**	**324**	**370**	**440**	**582**
	FJ936171	Reference	FPV	A	P	F	Y	Q	T	K
	GU569943	Reference	Original CPV-2	A	P	F	Y	Q	T	K
1	KY937668	canine/Guangzhou/P1-1/2016	CPV-2b	A	P	Y	I	Q	A	K
2	KY937669	canine/Guangzhou/P1-2/2016	CPV-2a	A	P	Y	I	Q	A	K
3	KY937670	canine/Guangzhou/P2/2016	CPV-2b	A	P	Y	I	Q	A	K
4	KY937671	canine/Guangzhou/P3/2016	CPV-2a	A	P	Y	I	Q	A	K
5	KY937672	canine/Guangzhou/P4/2016	CPV-2a	A	P	Y	I	Q	A	K
6	KY937674	canine/Guangzhou/T/2016	CPV-2a	A	P	Y	I	Q	A	K
7	KY968642	canine/Guangzhou/Z1/2016	CPV-2b	A	P	Y	I	Q	A	K
8	KY968643	canine/Guangzhou/Z2/2016	CPV-2b	A	P	Y	I	Q	A	K
9	KY937656	canine/Guangzhou/F2/2016	CPV-2b	A	P	Y	I	Q	A	K
10	KY937657	canine/Guangzhou/F3-1/2016	CPV-2b	A	P	Y	I	Q	T	K
11	KY937658	canine/Guangzhou/F3-2/2016	CPV-2c	A	P	Y	I	Q	A	K
12	KY937650	canine/Guangzhou/B/2016	CPV-2c	G	S	Y	I	Q	T	K
13	KY937647	canine/Shenzhen/11-1/2016	CPV-2c	A	P	Y	I	Q	T	K
14	KY937648	canine/Shenzhen/11-2/2016	CPV-2c	A	S	Y	I	Q	T	K
15	KY937662	canine/Shenzhen/H1/2016	CPV-2c	G	S	Y	I	R	T	K
16	KY937663	canine/Shenzhen/H4/2016	CPV-2a	A	P	Y	I	Q	A	K
17	KY937675	canine/Shenzhen/Y3/2016	CPV-2a	A	P	Y	I	Q	A	K
18	KY937673	canine/Shenzhen/S1/2016	CPV-2a	A	P	Y	I	Q	A	K
19	KY937660	canine/Guangzhou/GZ-4/2017	CPV-2a	A	P	Y	I	Q	A	K
20	KY937659	canine/Guangzhou/GZ-1/2017	CPV-2b	A	P	Y	I	Q	A	K
21	KY937661	canine/Guangzhou/GZ-5/2017	CPV-2a	A	P	Y	I	Q	A	K
22	MT488467	canine/Guangzhou/DH1130-1/2017	CPV-2c	A	P	Y	I	R	T	K
23	MT488468	canine/Guangzhou/DH1130-2/2017	CPV-2c	A	P	Y	I	R	T	K
24	MT488454	canine/Guangzhou/CWC/2018	CPV-2c	A	P	Y	I	R	T	K
25	MT488456	canine/Guangzhou/QH1/2018	CPV-2a	A	P	Y	I	Q	A	N
26	MT488457	canine/Guangzhou/QH2/2018	CPV-2a	A	P	Y	I	Q	A	K
27	MT488458	canine/Guangzhou/QH3/2018	CPV-2c	A	P	Y	I	R	T	K
28	MT488459	canine/Guangzhou/QH5/2018	CPV-2c	A	P	Y	I	R	T	K
29	MT488460	canine/Dongguan/XH1/2018	CPV-2c	A	P	Y	I	R	T	K
30	MT488461	canine/Dongguan/XH2/2018	CPV-2a	A	P	Y	I	Q	A	K
31	MT488462	canine/Dongguan/XH3/2018	CPV-2a	A	P	Y	I	Q	A	N
32	MT488463	canine/Shenzhen/XH4/2018	CPV-2a	A	P	Y	I	Q	A	K
33	MT488464	canine/Shenzhen/XH5/2018	CPV-2a	A	P	Y	I	Q	A	N
34	MT488465	canine/Guangzhou/DH6/2018	CPV-2c	A	P	Y	I	R	T	K
35	MT488466	canine/Guangzhou/DH7/2018	CPV-2b	A	P	Y	I	Q	T	K
36	MT488455	canine/Guangzhou/QG3/2019	CPV-2c	A	S	Y	I	R	T	K
37	MT488453	canine/Guangzhou/QG18/2019	CPV-2c	A	S	Y	I	R	T	K
38	MT488452	canine/Guangzhou/QG21/2019	CPV-2c	A	S	Y	I	R	T	K

### Phylogenetic analysis

**Figure 1:**
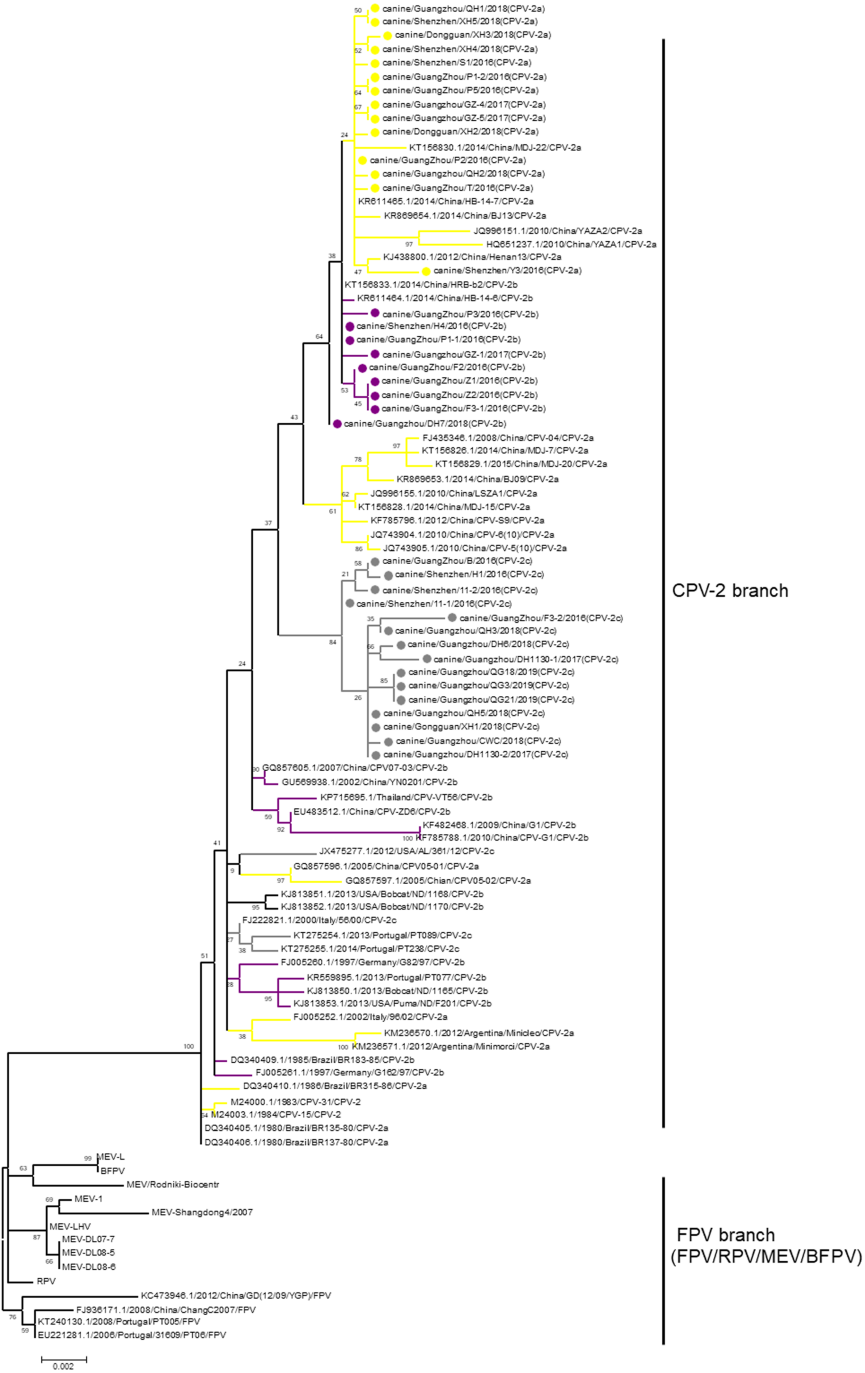
The phylogenetic analyses of VP2. Phylogenetic analyses were operated using the maximum likelihood method with 1,000 times bootstrap. The sequences uploaded in this study were labelled by a dot. Different CPV-2 variants were shown in different colours: Orange (CPV-2a), Purple (CPV-2b), and Gray (CPV-2c).

Phylogenetic analysis is a prominent method for studying the evolution of viruses. Phylogenetic analysis has been performed in many previous studies to elucidate the classification of CPV-2 (Geng et al., 2015; Wang et al., 2016a; Wang et al., 2016b). However, phylogenetic branches between different studies have been inconsistent, resulting in unclear evolution. The reason for the inconsistency among the studies is that the bootstrap values of the phylogenetic branches in those studies were low. For the current analysis, all available sequences of CPV-2, FPV, MEV, RPV, and BFPV in GenBank were obtained, and a phylogenetic tree was constructed with the neighbour-joining method with 1,000 bootstraps. Although all sequences of CPV-2 clustered closely together, the bootstrap values of the branches in this CPV-2 cluster were low. In fact, only the bootstrap value between CPV-2 and other viruses was 100. Therefore, we selected virus sequences from the neighbour-joining trees and used the maximum likelihood method to reconstruct a tree in which the CPV-2 cluster was separated from other viruses with a bootstrap value of 100 (Fig. 1). In the CPV-2 branch, the same variants did not cluster with each other, and the bootstrap values between the subbranches of CPV-2 were very low (Fig. 1). RPV clustered with FPV in one branch and BFPV with MEV in another branch, but the bootstrap values of these two branches were also low (Fig. 1). Hence, different animal parvoviruses derived from FPV were separated into only a CPV-2 branch and FPV branch, which includes FPV, RPV, MEV and BFPV. Additionally, all CPV-2 isolates belong to the CPV-2 branch (Fig. 1).

### Increasing prevalence of CPV-2c in China

**Figure 2:**
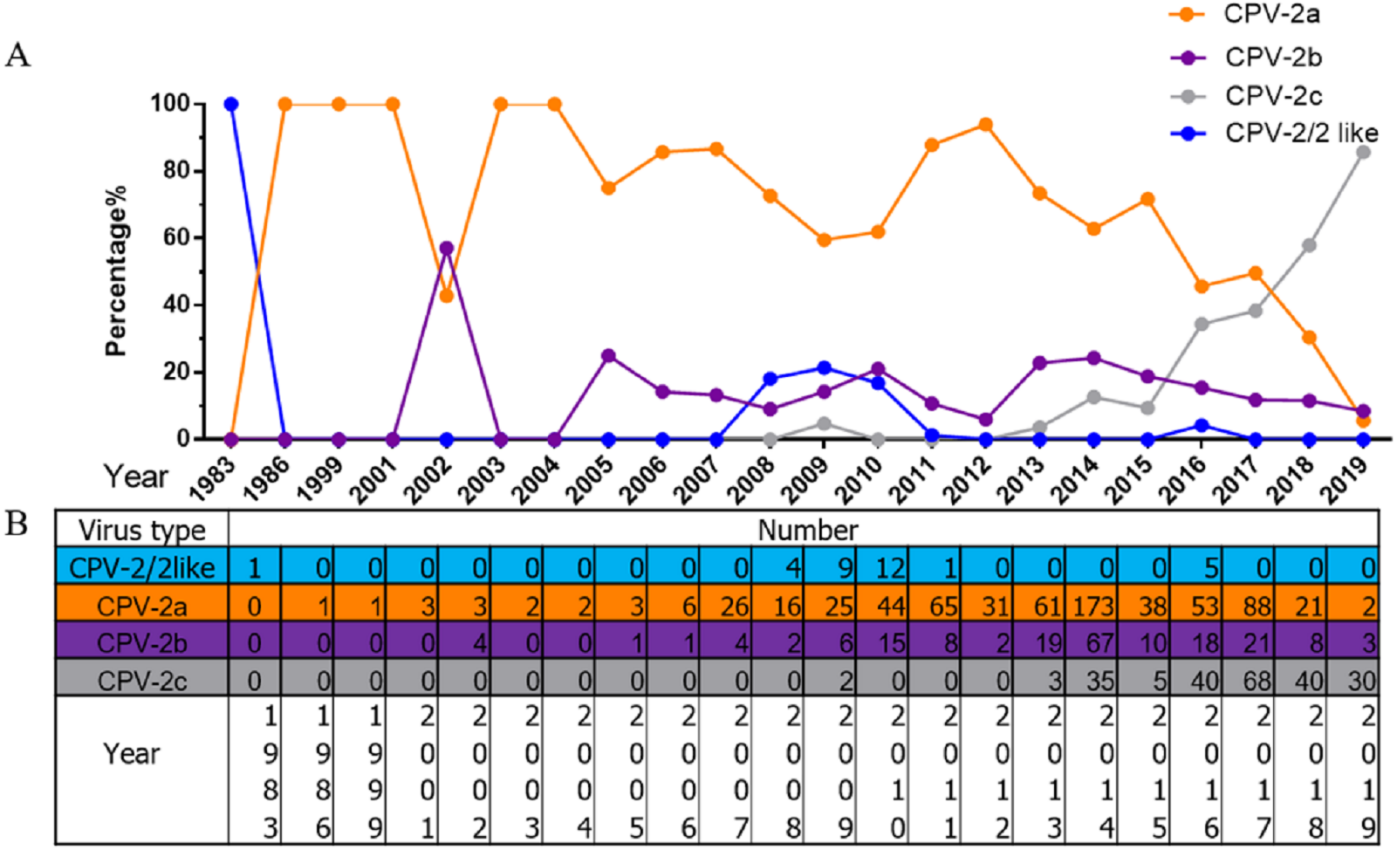
The percentages and numbers of different CPV-2 variants. The percentages (A) and numbers (B) of CPV-2 (includes the original CPV-2 and CPV-2-like strains), CPV-2a, CPV-2b, and CPV-2c strains.

All 1,076 Chinese CPV-2 isolates were classified and analysed according to collection date and variant type (Fig. 2). The percentages of different types of CPV-2 varied consistently over time. The original CPV-2 strain was sporadically present in China and generally regarded as a vaccine isolate (Zhang et al., 2010a; Zhang et al., 2010b). CPV-2a was the dominant strain until 2015; the prevalence of CPV-2a was highest in 2014, after which it showed a declining trend. CPV-2b first appeared in 2004 and had a low but stable prevalence until 2015, with a similar declining trend after 2014. It is worth noting that CPV-2c was not recorded until 2009; it has been increasing since. CPV-2c was on the rise before 2017 (Qi et al., 2020). In 2017–2018, CPV-2c predominated over CPV-2a and became the primary variant in China. CPV-2a and CPV-2b were replaced by CPV-2c in just a few short years.

## Discussion

As a virus with a DNA genome, CPV-2 is susceptible to mutation (Shackelton et al., 2005). Recently, the new variants CPV-2a, CPV-2b, and CPV-2c have gradually replaced the original CPV-2 (Ogbu et al., 2019), which mainly developed according to an aa mutation at site 426 in VP2 (N: CPV-2a, D: CPV-2b, E: CPV-2c). During evolution, CPV-2a/2b/2c, which not only infect dogs but also cats, completely replaced the original CPV-2, which infected only dogs (Ikeda et al., 2000; Parrish, 1991; Parrish et al., 1991; Perez et al., 2012; Truyen et al., 1996). Additionally, it has been reported that the appearance of novel variants of CPV-2 that affect both vaccinated and unvaccinated dogs is occurring (Calderon et al., 2011). Therefore, it is essential to investigate the evolution of CPV-2 in dogs. From 2016 to 2019, an aetiological survey was carried out in Guangdong, China, including Guangzhou, Shenzhen, and Dongguan cities.

Using PCR detection and virus isolation, 55.7% (34/61) of samples were identified as positive, which is a high rate, indicating that CPV-2 is a major canine enteric pathogen among dogs. CPV-2c is prevalent and predominates over CPV-2a in this regional aetiological survey in Guangdong. According to recent research in Vietnam, CPV-2c is more prevalent than other variants and shares an evolutionary origin with the Asian strain (Hoang et al., 2019). Hence, the prevention and treatment of CPV-2 disease should be given more attention in dog veterinary care.

In addition to multiple variants circulating in dogs, coinfection with variants and coinfection with other canine enteric pathogens in several cases were identified in this study. Moreover, coinfection with other pathogens can aggravate the disease (Zheng et al., 2018). These results explain the complex infection situation in CPV-2 disease, indicating that it is urgent to consider these factors in the prevention and treatment of CPV-2 disease.

Alanine at residue 297 discovered in this study is consistent with a 2017 report verifying that 297A has been predominant in CPV-2 after 1990 (Zhou et al., 2017). Furthermore, position 267 and 324 in all CPV-2 isolates was Y and I, respectively. It is worth noting that A5G and Q370R, which were recently reported in China, were also detected in some CPV-2c isolates from Guangdong. One hundred percent (38/38) presented 324I, and 57.89% (22/38) presented 440A, which may indicate that the mutated strains are adapting to dogs. Moreover, several newly identified CPV-2c strains carried mutations at residue 13, and among other CPV-2c variants, more conservative P residues were common. The P13S residue was found in European cats (Decaro et al., 2008) and Japanese dogs (Nakamura et al., 2004), and in China, the same mutation occurred in CPV-2 isolates (accession numbers: GU392236, GU392244, GU392243, GU392242, GU392241, KM083037, KM083038, KJ170679) from raccoon dogs and foxes in 2009. A mutation in K582N was observed in this investigation. Whether these mutated amino acids have an effect on viral evolution deserves further study.

It is essential to note that the phylogenetic analysis was inconsistent with those in previous studies, resulting in unclear evolution. After performing the phylogenetic analysis of all sequences representing all viruses derived from FPV, we concluded that these viruses are separated into only CPV-2 and FPV branches, which includes FPV, RPV, MEV and BFPV. Clarification of the phylogeny would be useful for virus evolution research.

Although a previous study indicated that CPV-2a remains the major variant in China, accounting for 61.81% of CPV-2 isolates (Zhuang et al., 2019), with the rise of an increasing number of CPV-2c isolates, CPV-2c has to some extent become the dominant variant in China. A recent review showed that CPV-2c had been on the rise before 2017 (Qi et al., 2020). However, according to our research, CPV-2c may have become the main variant of CPV-2 in China. CPV-2c appears to have succeeded in replacing other variants as the major strain in only 10 years. This situation is similar to that in neighbouring Vietnam. The first CPV-2c strain in Vietnam was reported in 2004, after which it gradually evolved into the predominant variant (Hoang et al., 2019). The first Chinese CPV-2c isolates were collected in only 2009, and the evolution of CPV-2c in China lagged behind that in other regions. CPV-2c appears to be present in many countries worldwide, which will be a great challenge for disease control. Notably, the most recently used vaccines in China are mainly CPV-2 (Nobivac, Holland) (Wang et al., 2016b), and whether this type of vaccine still confers full protection against novel variants is unclear. Vaccine immunity protects most dogs from CPV infection, but vaccine protection can fail. Some vaccinated dogs may still be re-infected with canine parvovirus, and the vaccine may not provide complete protection against some endemic strains (De et al., 2018; Wu et al., 2015). Therefore, continued monitoring for CPV-2 prevalence and effective vaccines are required in the future.

## Conclusions

A recent aetiological survey conducted in Guangdong, China, showed that 34/61 dogs with diarrhoea tested positive for parvovirus. Among them, 38 specific VP2 sequences were amplified. The function of several newly discovered variant sites deserves further study. It is worth noting that CPV-2c infection in domestic dogs in China is increasing. Based on this analysis, CPV-2c has replaced CPV-2a as the dominant variant. The results of this study provide fundamental knowledge regarding CPV-2 strains circulating in China.

##  Supplemental Information

10.7717/peerj.9869/supp-1 File S1 All the VP2 sequences in this study

## Additional Information and Declarations

### Data Availability

The following information was supplied regarding data availability: The VP2 sequences are available at GenBank: KY937668; KY937669; KY937670; KY937671; KY937672; KY937674; KY968642; KY968643; KY937656; KY937657; KY937658; KY937650; KY937647; KY937648; KY937662; KY937663; KY937675; KY937673; KY937660; KY937659; KY937661; MT488467; MT488468; MT488454; MT488456; MT488457; MT488458; MT488459; MT488460; MT488461; MT488462; MT488463; MT488464; MT488465; MT488466; MT488455; MT488453; MT488452.
